# Unveiling the Dual Threat: How Microbial Infections and Healthcare Deficiencies Fuel Cervical and Prostate Cancer Deaths in Africa

**DOI:** 10.3390/pathogens13030243

**Published:** 2024-03-10

**Authors:** Sofian Abdul-Nasir, Hyungdon Lee, Md. Habibur Rahman, Johny Bajgai, Kyu-Jae Lee, Cheol-Su Kim, Soo-Ki Kim

**Affiliations:** 1 Department of Convergence Medicine, Wonju College of Medicine, Yonsei University, Wonju 26426, Republic of Korea; abdulnasirsofian62@gmail.com (S.A.-N.); medbio@yonsei.ac.kr (K.-J.L.); cs-kim@yonsei.ac.kr (C.-S.K.); 2 Department of Internal Medicine, College of Medicine, Hallym University, Chuncheon Sacred Heart Hospital, Chuncheon 24253, Republic of Korea; 3 Research Institute of Metabolism and Inflammation, Wonju College of Medicine, Yonsei University, Wonju 26426, Republic of Korea

**Keywords:** cervical cancer, prostate cancer, bacteria, virus, Africa, mortality, healthcare

## Abstract

Cervical and prostate cancer account for 7.1 and 7.3 deaths per 100,000 people globally in 2022. These rates increased significantly to 17.6 and 17.3 in Africa, respectively, making them the second and third leading cause of cancer deaths in Africa, only surpassed by breast cancer. The human papillomavirus is the prime risk factor for cervical cancer infection. On the other hand, prostate cancer risks include ageing, genetics, race, geography, and family history. However, these factors alone cannot account for the high mortality rate in Africa, which is more than twice the global mortality rate for the two cancers. We searched PubMed, Embase, Scopus, and Web of Science to select relevant articles using keywords related to microorganisms involved in cervical and prostate cancer and the impact of poor healthcare systems on the mortality rates of these two cancers in Africa by carrying out a detailed synopsis of the studies on microbial agents involved and the contributory factors to the deteriorating healthcare system in Africa. It became apparent that the developed countries come first in terms of the prevalence of cervical and prostate cancer. However, more people per capita in Africa die from these cancers as compared to other continents. Also, microbial infections (bacterial or viral), especially sexually transmitted infections, cause inflammation, which triggers the pathogenesis and progression of these cancers among the African population; this has been linked to the region’s deficient health infrastructure, making it difficult for people with microbial infections to access healthcare and hence making infection control and prevention challenging. Taken together, untreated microbial infections, primarily sexually transmitted infections due to the deficient healthcare systems in Africa, are responsible for the high mortality rate of cervical and prostate cancer.

## 1. Introduction

Cancer-related mortality remains a worrying concern in public health, accounting for approximately 10 million deaths annually [1]. Of all cancer-related deaths, cervical cancer (CC) and prostate cancer (PCa) are the sixth and fifth leading causes of cancer mortality globally but third and second in Africa, respectively [1]. Except for breast cancer, CC and PCa cause more deaths than other cancer types in Africa [2]. In the global context, CC and PCa account for 7.1 and 7.3 deaths per 100,000 people globally in 2022 [1]. Several risk factors account for the etiology of these cancers. These factors include microbial infections, such as the human papillomavirus (HPV), with which CC is primarily associated. At the same time, age, race, geography, family history, and genetics are considered the predominant causative factors of PCa [3,4]. 

**Figure 1:**
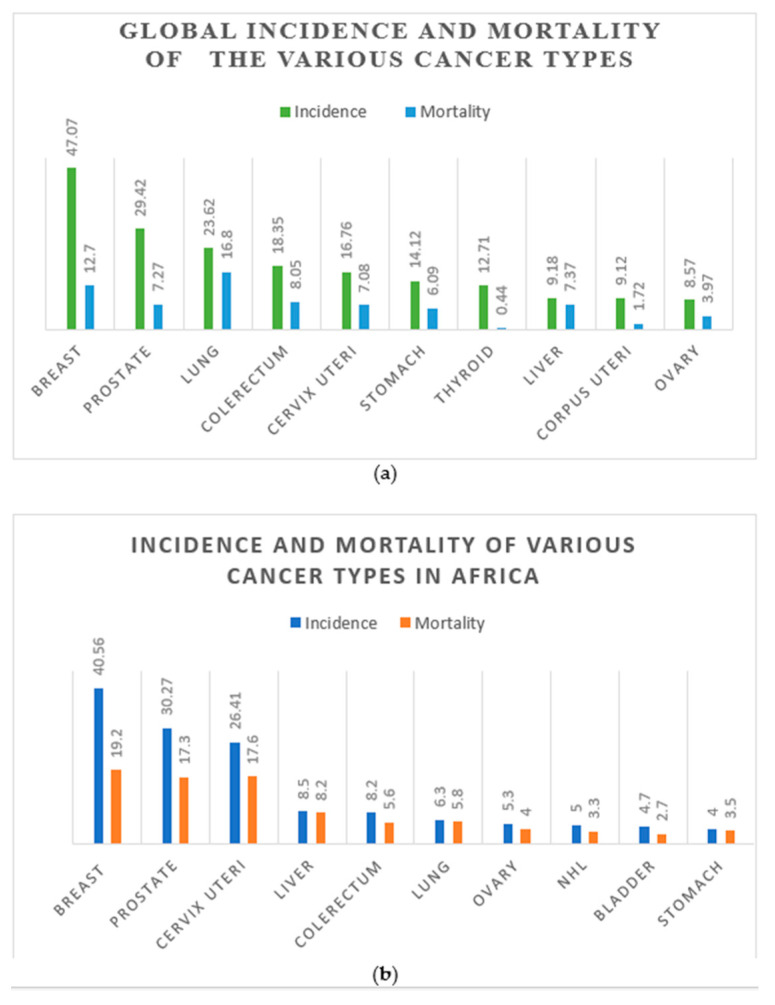
The distribution of global cancer incidence and mortality rates in 2020 (**a**). The distribution of cancer incidence and mortality rates in Africa in 2020 (**b**). Numbers are given per 100,000 people. (Data source: [1]).

Despite reducing incidence and mortality rates for these cancers in some developed countries, the picture looks different in Africa. Even more worrying is that the mortality rates, respectively, increased marginally to 17.6 and 17.3 in Africa, which exceeded the world mortality rates by more than 2-fold (CC; 2.5-fold and PCa; 2.4-fold) (Figure 1a,b) [1].

Therefore, the driving forces that orchestrate the high mortality of patients with these cancers in Africa should be re-examined. In this narrative review, we searched PubMed, Embase, and Web of Science to select relevant articles using key words such as ‘cervical cancer’ AND ‘prostate cancer’ AND bacteria AND virus AND Africa AND ‘healthcare system’ AND mortality. We also synthesized data from the World Health Organization’s (WHO) website, together with the relevant literature, to elucidate the mechanisms linking microbial infections, poor healthcare systems, and the high mortality rates of CC and PCa in Africa. In the first section of this review, we briefly highlight the pathophysiology and epidemiology of CC and PCa. The second section discusses the microorganisms involved in the pathogenesis and mortality of these two cancers. The third section discusses the factors that contribute to the poor healthcare system in Africa, how they affect health delivery, and link to the high mortality of CC and PCa in Africa.

## 2. Pathophysiology and Epidemiology of Prostrate and Cervical Cancer

### 2.1. Prostate Cancer

The prostate gland, an androgen-stimulating organ whose secretion forms part of the semen, is the target site of PCa, and mutations in the glandular cells that constitute the prostate may orchestrate nodule formation and PCa [5]. This tumor may spread to the bone or lymph nodes or remain inside or close to the nearby prostatic tissue [6]. Most PCa cases are diagnosed as localized illnesses, which are typically asymptomatic [7]. In these circumstances, abnormal prostate-specific antigen (PSA) and abnormal digital rectal exam (DRE) levels may be the earliest indicators of malignancy, thus providing a chance for prompt intervention [8]. Nonspecific lower urinary tract symptoms associated with PCa include nocturia, hematuria, dysuria, and sexual dysfunction. Also, bone pain, most commonly in the vertebrae, pelvic region, ribs, or proximal femur, erectile dysfunction, weight loss, urine retention or incontinence, and weakness, among other symptoms, may be experienced by patients with metastatic PCa [9].

PCa is ranked the second most common cancer among men, which affects one in eight men in their lifetime, per the WHO Global Cancer Observatory (GLOBOCAN) data [1]. The prevalence of metastatic PCa at presentation ranged between 6.3% and 8% [10]. Furthermore, around 15% of patients with localized PCa at presentation treated curatively advanced to metastatic disease [11]. The significant risk factors of PCa include age, race, genetics, geography, and family history [3,12]. The above risk factors are ‘clear risk factors’ according to the American Cancer Society. However, diet, obesity, smoking, chemical exposure, STIs, and vasectomy are considered ‘less clear risk factors’ of PCa [13]. Men in the age range of 50–70 have a high likelihood of developing PCa. Moreover, elderly white US adults between the ages of 75 and 79 have more than 100 times the chance of developing PCa relative to 45–49-year-olds [4]. In terms of race and geographic location, Australia, Europe, and North America have the highest incidences of prostate cancer, i.e., the top three globally [1].

In contrast, the lowest incidences are found in Africa, Asia, Latin America, and the Caribbean [1]. Genetically, mutations in BRCA1 or BRCA2 pose a risk. Men with the androgen receptor gene, which contains a polymorphic region of CAG repeats with less than 18 in length, have a higher likelihood of developing prostate cancer compared to those with 26 repeats or longer; this explains why black Americans and people of African origin have high PCa risks [13,14,15]. In the family tree, men whose close relatives, such as father, brother, or son, have cancer of the prostate also have a higher chance of developing it as well [15].

On a global scale, from 2000 to 2022, the mortality of PCa declined, but with an increasing incidence rate, probably because of PSA screening and treatment [1]. However, the risk of mortality is higher among Africans affected by CC and PCa [2]. As shown in Figure 1b, the GLOBOCAN data for 2022 showed that the mortality of CC and PCa in Africa is more than double the global rate (Figure 1a,b). Also, it has been conclusively reported that PCa might be underreported or underdiagnosed in Africa; however, its incidence and mortality are still a serious public health concern [16]. Epidemiological and surveillance studies also reveal a high mortality rate of PCa among black people relative to their white counterparts, as proven by a comparative study between black people in West Africa and America, which confirmed a similar PCa incident rate [17]. 

In this section, the evidence reviewed suggests that the developed countries of Europe, Australia, and North America have the highest morbidity of PCa. Surprisingly, the mortality rate tells a different story, where lower-income countries like Africa have the highest death rates. Screening for early detection coupled with treatment options currently available, such as surgery, radiotherapy, hormone therapy, chemotherapy, and immunotherapy, among others, can help reduce the risk of PCa and consequently reduce the number of deaths [13].

### 2.2. Cervical Cancer

On the other hand, CC is a female disease and is considered among the fourth most dominant cancer that affects women [18]. It affects the cervix, specifically the squamous junction consisting of reserve cells above the basement membrane and the cervical epithelium, which are susceptible to malignant HPV [19]. HPV is a double-stranded DNA virus with over 450 genotypes, and almost all cancer cases contain 1 of 13 malignant genotypes of HPV [20]. It also includes approximately 150 types, and is considered the most significant risk factor for CC [21]. However, factors such as smoking, sexual history, chlamydia infection, weak immune system, use of oral contraceptives, multiple full-term pregnancies, low fruit or vegetable diet, taking diethylstilbestrol, and having a family history of CC are also regarded as potential risk factors [13].

The marker of HPV infection is a growth (warts) referred to as papilloma on the surface of the anus, genitals, mouth, and throat; hence, can spread by skin contact through sex: oral, vagina, or anal [13]. Genital warts are non-carcinogenic; thus, are classified as a ‘low-risk’ HPV type, which is caused by types 6, 11, 42, 43, and 44 [21]. However, the carcinogenic HPV strains ‘16, 18, 31, 33, 34, 35, 39, 45, 51, 52, 56, 58, 59, 66, 68, and 70’ can alter tissues in the cervix into malignant tissues upon initial HPV infection—this leads to lesions call intraepithelial neoplasia [22,23]. Further, HPV genotypes also exhibit regional variability. For instance, genotype 35 has been linked to a high risk of CC among individuals of African descent as compared to other racial groups [24].

Irrespective of the genotype, any form of HPV infection should be considered virulent until confirmed otherwise. An HPV infection can be active without visible or microscopic alterations of the cervix and mostly disappears in 1 to 2 years as a result of suppression by the host immune system or biological unfitness [25]. Despite evidence of immunity against secondary infection, immunity after natural infection is yet to be fully understood. In contrast, the HPV vaccine can provide approximately 90% immunity against the disease, lasting about 15 years [26]. 

CC is a complicated disease, and HPV is the main initiating factor. Thus, understanding the intricate interplay of the virus, host genetics, and cellular processes involved in transformation is crucial to developing effective preventive, diagnostic, and therapeutic strategies.

Just like PCa, the morbidity and mortality rate of CC in Africa is also on an upward spiral, with about 34 out of every 100,000 women infected and about 70% mortality (relative to approximately 5% in developed countries) [22]. Screening and vaccination are effective in preventing CC. However, available treatment modalities currently include surgery, radiotherapy, immunotherapy, chemotherapy, and targeted drug therapy [13,27]. 

The incidence and mortality of CC in Africa is approximately 80%. However, no evidence reviewed above indicates the cause of the high mortality rate; hence, further interrogation is needed. The following section discusses the microorganisms involved in CC and PCa.

## 3. Microbial Agents and Cancer

Aging, geography, ethnicity, and genetics, among others, as already noted above, are particularly predominant factors that predispose a person to cancer. However, microbial pathogens such as bacteria and viruses are implicated in cancers of the prostate and cervix. Approximately 20% of cancer incidences are caused by infectious agents, and a good number of studies have shown the presence of some bacterial and viral agents such as *Escherichia coli*, *Cutibacterium acnes*, *Neisseria gonorrhea*, HPV, *Herpes simplex*, Epstein–Barr virus, and *Mycoplasmas* [28,29]. Lawson et al. (2022) concluded in their pooled studies that, except for HPV, the microorganisms mentioned earlier have roles in PCa oncogenesis, which is yet to be proven. They also hinted at the potential but unknown roles of *Cytomegalovirus*, *Chlamydia trachomatis*, *Trichomonas vaginalis*, and *Polyomaviruses* in chronic prostatic inflammation of the prostate [28]. Herpes Simplex Virus types 1 and 2, HPV, Human Herpes Virus 8, *Cytomegalovirus*, and Hepatitis C (HCV) have been found in the biopsies of CC and PCa patients [30]. Also, about 90% of all CC cases are instigated by HPV [31].

Most of these microbial agents involved in cancer pathogenesis are usually part of the normal microflora, involved in maintaining good gut health, that become virulent due to a breach in any part of the surface, immune weakness, or incomplete antimicrobial therapy [32,33]. Even though it is expected that a harmonious balance exists between the host and its microbiota, the relationship between these active organisms and urogenital health has yet to be determined [32]. That notwithstanding, various agents, for example, drugs, environmental factors, and exogenous pathogenic bacteria, may offset the balance and eventually instigate multiple disease conditions, including cancer [33]. Thus, the habitat of human-dwelling microbes, including the biotic and abiotic components constituting the microbiome, could directly or indirectly influence the various stages of cancer either at the site of carcinogenesis or by regulating changes in metabolism and immunity [34].

The effects of these infectious microorganisms on cancer patients can be adverse if not treated. Research reports showed that close to 90% of cancers in developed countries are diagnosed before they become out of control, as compared to about 30% in developing countries, due to efficient and underdeveloped healthcare systems, respectively [2,35]. Therefore, transmission of microbial infection tends to cause CC and PCa if immediate and prompt infection treatment is not provided. Further details of specific bacteria and viruses commonly implicated in CC and PCa pathogenesis and spread are discussed below.

### 3.1. Bacteria Species in Cervical and Prostate Cancers

Even though the role of microbial agents in cancer etiology has long been acknowledged, it took some time before the idea became established [31]. Tissue culture of the prostate from about 64 men undergoing prostatectomy identified more than 85 microorganisms noted to be involved in chronic inflammation of the prostate in some studies carried out between 2005 and 2010, where the *Propionibacterium* spp. was reported to be the most prominent strain [36,37]. Caini et al. (2014) observed that people who have previously been infected with gonorrhea have a 20% risk of developing PC and that men infected with PC had bacteria in their prostate tissues, with *Neisseria gonococcus* being the most common bacterial species identified [30]. Other studies using 16S rDNA sequencing and PCR to examine the urine and prostate biopsies of PCa patients established the presence of *Bacteroides massiliensis*, *Streptococcus*, *Bacteroides* spp., *Corynebacterium*, *Staphylococcus*, *Pseudomonas*, *Escherichia coli*, Acinetobacter, *Helicobacter pylori*, *Gardnerella vaginalis*, and Propionibacterium in the samples [36,38,39]. These microbes could be involved in initiating or promoting PCa progression through inflammation.

On the other hand, the number of bacterial species involved in CC includes the following: *Lactobacillus*, *Campylobacter*, *E. coli*, *Klebsiella pneumoniae*, *Enterococcus faecalis*, *Proteobacteria*, *Enterobacter cloacae*, *Pseudomonas aeruginosa*, *Morganell amorganii*, and *Enterobacter aerogenes* [40,41]. Also, bacterial genera such as *Sneathia*, *Gardnella*, *Atopobium*, *Prevotella*, *Ureaplasma*, *Bacteroides*, and *Leptotrichia*, which exist as part of the gut or vaginal microbiome, have been identified at higher levels in patients with cervical lesions [32]. All these bacteria increase the risk of CC as well as PCa. They can also serve as biomarkers for identifying ulcerations in the cervix as well as identifying HPV and CC risk in women [42]. 

Even though some reports ruled out the possibility of other microbial involvement in PC, except for *N. gonococcus*, in the face of this overwhelming evidence, it will be sound to infer that those bacteria (especially sexually transmitted infection (STI) strains) play a crucial role in PCa progression and mortality. Other studies reported that Chlamydia trachomatis and Trichomonas vaginalis could also be involved in CC and PCa. Evidence suggests that STIs can increase the risk of PCa by inducing chronic inflammation within the prostatic tissue, thereby orchestrating uncontrolled cell proliferation and consequently resulting in carcinogenesis [29,43]. In addition, there have been suggestions that a history of multiple STI infections or untreated infections could result in a higher possibility of PCa development [44]. Also, some studies established that Mexican, American, and Asian people with a history of STIs are more prone to developing PC as compared to those without a previous STI history. Whilst Cheng et al. (2010) expressed a high probability of STIs in PCa oncogenesis, Vazquez-Salas et al. (2016) explicitly stated that people with a gonorrhea infection history are two times more likely to develop cancer of the prostate [45,46].

Moreover, an observation made by a study showed that *E. coli*, *Klebsiella pneumoniae*, *Enterococcus faecalis*, *Proteobacteria*, *Enterobacter cloacae*, *Pseudomonas aeruginosa*, *Morganell amorganii*, and *Enterobacter aerogenes* were found in varying percentages in the discharges of CC patients, with *E. coli* being the dominant microbe (approximately 62.92%) [40]. They concluded that *E. coli* and HPV coinfection contribute to CC development. Thus, STIs such as gonorrhea, syphilis, Trichomonas vaginalis, and Chlamydia trachomatis are among the dominant bacterial species noted for initiating and facilitating CC and PC carcinogenesis and metastasis.

The high prevalence of these pathogenic bacteria in CC and PCa patients might be the driving force of inflammation of the cervix and prostate, resulting in progression and severity, respectively [29,43]. Recent work in chronic inflammation asserts that microorganisms are implicated in the pathogenesis and progression of cancer since inflammation drives about 20% of cancer incidence [28]. Others also believe that some microbes, such as *H. pylori*, interfere with cell cycle regulation, resulting in uncontrolled proliferation, which is characteristic of all cancers, enabling PCa tumorigenesis [47]. Also, immune-associated gene downregulation and suppressing immune cell expression are the mechanisms by which *Gardnerella vaginalis* induces cancer [33].

The inflammation, disruption in the cell cycle, and downregulation of the immune system caused by microbes can increase the risk of mortality. The spike in mortality cannot only be attributed to bacteria involvement; viruses also play a role in cancer pathogenesis and mortality, which are discussed in the next section.

### 3.2. Viruses Implicated in Cervical and Prostate Cancer

Some viral strains, like bacteria, have been associated with CC and PCa infection viz Herpes Simplex Virus types 1 and 2, Human Herpes Virus 8, HPV, Cytomegalovirus, and Polyomaviruses [28,48,49]. More than 90% of all CC cases have been reported to be caused by HPV [49].

The primary mechanism by which these viral pathogens drive the pathogenesis and progression of CC and PCa is inflammation induction in the cervix and the prostate that might be involved in destroying immune cells in the body, stifling their ability to check and destroy abnormal cell growth. Thus, these cells grow out of control and become malignant, leading to fatality [29,50]. Further, Gao et al., 2023, reported that the F-box protein—FBXO22—enhances HPV-linked CC proliferation and reduces autophagy by blocking the liver kinase B/AMPK signaling [51]. The fatalities of these cancers connected with viral sources are unusually high in developing countries like Africa, who are still battling with infection prevention. More details on viruses involved in the two cancers under consideration are summarized in good reviews found here [52,53].

### 3.3. Other Factors Involved in Cervical and Prostate Cancers

Apart from the factors noted above, other risk factors for CC and PCa include smoking, long-term use of hormonal contraceptives, poor diet, immunosuppression, promiscuity, and HIV infection [49,54]. Among these, smoking has been linked to HPV infection and progression, resulting in high CC incidence [55]. Related to smoking are environmental agents, some of which include chemicals contained in cigarette smoke like coal tar, smoke inhaled from burning wood, and tar-based sanitary pads that induce signaling pathways suitable for HPV-related cervical carcinogenesis [56]. The WHO has established that most inhabitants of developing countries such as Africa depend on crop residue, wood, and animal dung for cooking and heating. Since biomass stoves are noted for bio-carcinogen emission, women become exposed to smoke from these stoves and hence stand higher chances of developing CC [57,58].

Further evidence suggests that these cancers have other unusual etiological factors. For instance, working in certain occupations, namely military/law enforcement, farming, management, administrative jobs, public safety, night shift work, and toxic substances in some work environments may have elevated risks for PC [52,59,60]. Additionally, people employed in managerial and military occupations are at 2 and 3 times the risk of developing PCa overall and aggressive PCa, respectively, as compared to the usual occupations of Ghanaian men, who have very low rates of PSAs in screenings [59]. However, some of these studies are limited to individuals with European ancestry who have high rates of PSAs in screenings, which can lead to biased results. Thus, in brief, hormone-based contraceptives, sexual promiscuity, HIV infection, smoking, exposure to smoke, and working in specific jobs can also increase the risk of CC and PC and thus could be partly responsible for more people with these cancers dying.

## 4. Factors That Militate against Good Healthcare Delivery in Africa 

Poor healthcare systems in Africa have negatively impacted healthcare delivery efforts. One of the contributory factors is the lack of capacity to control infections due to inadequate infrastructure, such as electricity supply, running water, and insufficient sanitation measures [61]. Water is *sine qua non* to infection control and an efficient operation of healthcare facilities [62]. Hence, the erratic water supply can dwindle the efforts to control infections, making it difficult to provide adequate patient care and implement infection control measures such as proper disposal of clinical waste and hand sanitization [63]. 

Similarly, the need for more sustainable power is one of the infrastructural challenges causing poor healthcare management in Africa. An analysis of healthcare service provision and access to energy in 2012 and 2013 in Senegal showed that less than 50% of health facilities have access to electricity, with 18% and 3% of facilities using fuel-powered generators and solar systems, respectively [64].

In addition, other factors, such as insufficient budgetary allocation, scanty human resources, poor management and leadership, and corruption, have contributed to the African continent’s inefficient healthcare plight [65]. Inadequate budgets for health facilities can cause a shortage of resources, such as medical supplies and equipment, in many African hospitals. For instance, poor infrastructure or lack of resource availability, common in low- and middle-income countries (LMIC) to which Africa belongs, has been linked to an increased burden of CC [35]. An estimated 85% of worldwide cases of CC come from the LMIC, with a death rate of 70%, as compared to high-income countries with about 5%. The WHO grouped most parts of Africa, Eastern, Southern, and Middle, among the high-risk regions for cervical cancer [66]. Similarly, Uganda, in Africa, has the highest PCa mortality rate, and Sub-Saharan Africa leads globally in terms of cancer-related deaths, of which specifically cervical and prostate cancer are among [2,66].

Further, there is also a shortage of trained healthcare workers, epidemiological expertise, and resources for research, which have also been partly responsible for the current status quo of health in Africa [67]. These make it challenging to provide adequate supervision and training in infection control [65].

Moreover, low health insurance coverage, especially for the poor and vulnerable, is another significant factor that makes Africa’s healthcare systems less effective [68]. In a study by Barasa et al. (2021), only four countries in Sub-Saharan Africa had health insurance systems with more than 20% coverage, which include Rwanda, Ghana, Gabon, and Burundi. They also reported that most subscribers were people of rich backgrounds [69].

It is safe to infer that financial constraints result in inadequate budgetary allocation and infrastructure shortage, which affects the supply of the necessary resources such as power and water for health facilities. Also, bad management or lousy leadership in the government and health facilities opens doors to corruption. Hence, the depletion of the meager resources makes it challenging to fulfill most of these obligations, including providing health insurance for people experiencing poverty. All these will consequentially contribute to an increase in the disease burden, including cancer. An increase in the incidence of these cancers might not lead to deaths. However, when the necessary preventive and control measures are lacking because of the factors discussed above, it can cause an increase in mortality. For instance, some reports show that only close to 30% of all cancer cases in Africa are detected before they get to a point where treatment is no longer possible [35]. 

Even though a few areas in parts of Africa may have relatively good medical facilities, sociocultural beliefs could hinder access to healthcare. For example, fear, embarrassment, and lack of support from spouses are impediments to cervical screening exercises, as reported by Srinath et al. (2023) in a study on the barriers to CC and breast cancer screening in LMIC. They assessed availability, approachability, acceptability, affordability, awareness, and appropriateness. They found that the significant obstacles to screening were the need for awareness, the high cost of screening, and the distance from their places of dwelling to screening centers [70]. They emphasized the need to understand the risk factors and improve confidence in the health system. The differences in the effectiveness of intervention programs, such as screening and vaccinations between developed and developing countries, make preventable HPV-induced CC challenging to control in the latter [71].

**Figure 2:**
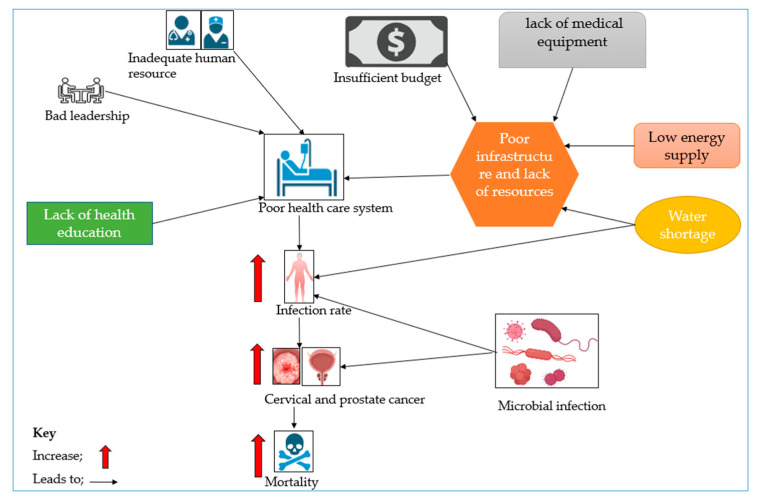
A scheme linking contributory factors to poor healthcare systems and to increases in CC and PCa incidences and mortality.

One potential factor linking microbial infection, CC and PCa, and poor healthcare systems in Africa is STIs, as noted under the infection agents in the cancer section. In Africa, STIs are prevalent due to multiple factors, including lack of health education, limited access to healthcare, and cultural factors that discourage people from talking about sexual health [72]. Further, lack of access to preventive measures such as testing and vaccinations, for instance, for HPV, as well as limited treatment options, can exacerbate the problem [73]. Poor healthcare delivery in Africa can also lead to delays, misdiagnoses and inadequate treatment for STIs and cancers, culminating in poorer health outcomes for individuals affected, thereby resulting in death, as illustrated in Figure 2. Together, inadequate human resources, low budgetary allocation, poor infrastructure, lack of health education, and bad leadership contribute to a flawed healthcare system, which is incapable of providing the necessary services like infection control; this results in increased infection rates contributing to CC and PCa, leading to an elevated mortality rate.

Microbial infection can also become rampant and contribute to the increase in the incidence of disease; this can lead to an increase in CC and PCa incidences and eventually more deaths. 

## 5. Conclusions

Considering these insights, it will not be out of place to conclude that microbial infections, which are primarily STIs, are the main drivers of the high mortality rate of cervical cancer and prostate cancer in Africa. Poor healthcare systems worsen the condition of the patients of these cancers since timely diagnosis and treatment cannot be possible because of poor health facilities. These findings imply that combating microbial disease can reduce the number of deaths associated with CC and PCa, which is only feasible by providing proper healthcare through an improved medical care system. Therefore, improving access to preventive measures and testing, improving health education, and strengthening healthcare systems through increased budgetary allocation, reliable power and water supply to health facilities, and good leadership is imperative. Together, these will mitigate the impact of microbial infections and reduce the incidence and mortality associated with CC and PCa.
